# Supplementary material for: End Sequence Analysis Toolkit (ESAT) expands the extractable information from single-cell RNA-seq data
Source: Genome Res. 2016 Oct;26(10):1397–410. doi: 10.1101/gr.207902.116 (PMC5052061; doi:10.1101/gr.207902.116)
Supplement: Supplemental Material [file supp_gr.207902.116_Supp_Materials.tar.gz › ESAT-esat_v0.1/doc/html/classbroad_1_1core_1_1motif_1_1_position_weight_matrix-members.html]

ESAT: Member List


ESAT


- Main Page
- Related Pages
- Packages
- Classes
- Files

- Class List
- Class Index
- Class Hierarchy
- Class Members

All Classes Namespaces Files Functions Variables Enumerator Pages

- broad
- core
- motif
- PositionWeightMatrix

broad.core.motif.PositionWeightMatrix Member List

This is the complete list of members for broad.core.motif.PositionWeightMatrix, including all inherited members.

|  |  |  |
| --- | --- | --- |
| addColumn(double[] site) | broad.core.motif.PositionWeightMatrix |  |
| addPseudoCounts() | broad.core.motif.PositionWeightMatrix |  |
| addToCentroid(Matrix m, PositionWeightMatrix pwm) | broad.core.motif.PositionWeightMatrix | protected |
| centroidOf(Collection< PositionWeightMatrix > pwmSet) | broad.core.motif.PositionWeightMatrix |  |
| computeScoreDistribution(float backgroundA, float backgroundC, float backgroundG, float backgroundT, int sampleSize) | broad.core.motif.PositionWeightMatrix |  |
| copy() | broad.core.motif.PositionWeightMatrix |  |
| createIsoPWM(double[] column, String name) | broad.core.motif.PositionWeightMatrix |  |
| distanceFrom(PositionWeightMatrix other) | broad.core.motif.PositionWeightMatrix |  |
| flatWeightMatrix(double infoTh) | broad.core.motif.PositionWeightMatrix |  |
| getAlignDir() | broad.core.motif.PositionWeightMatrix |  |
| getConsensus() | broad.core.motif.PositionWeightMatrix |  |
| getLeftHighInfoStart() | broad.core.motif.PositionWeightMatrix |  |
| getLogLikelihood(char[] s) | broad.core.motif.PositionWeightMatrix |  |
| getLogLikelihood(int[] s) | broad.core.motif.PositionWeightMatrix |  |
| getLogLikelihood(short[] encodedSequence, int i) | broad.core.motif.PositionWeightMatrix |  |
| getName() | broad.core.motif.PositionWeightMatrix |  |
| getNextKmer(int[] kmer) | broad.core.motif.PositionWeightMatrix | static |
| getNumCol() | broad.core.motif.PositionWeightMatrix |  |
| getNumEndPosToErase(double infoThreshold) | broad.core.motif.PositionWeightMatrix |  |
| getNumStartPosToErase(double infoThreshold) | broad.core.motif.PositionWeightMatrix |  |
| getRightHighInfoStart() | broad.core.motif.PositionWeightMatrix |  |
| ic() | broad.core.motif.PositionWeightMatrix |  |
| isEmpty() | broad.core.motif.PositionWeightMatrix |  |
| kullbackLeiber(PositionWeightMatrix other) | broad.core.motif.PositionWeightMatrix |  |
| match(SequenceRegion region, double[] background, float minScore) | broad.core.motif.PositionWeightMatrix |  |
| permuteColumns(boolean preserveGCDinucleotides) | broad.core.motif.PositionWeightMatrix |  |
| PositionWeightMatrix(String name) | broad.core.motif.PositionWeightMatrix |  |
| printKmer(int[] kmer) | broad.core.motif.PositionWeightMatrix |  |
| printRefinedWeightMatrix(double infoThreshold) | broad.core.motif.PositionWeightMatrix |  |
| reverseComplement() | broad.core.motif.PositionWeightMatrix |  |
| setAlignPos(int minAlignNum) | broad.core.motif.PositionWeightMatrix |  |
| setMatrix(List< double[]> sites) | broad.core.motif.PositionWeightMatrix |  |
| setMatrixFromRawData(List< String[]> unparsedRows) | broad.core.motif.PositionWeightMatrix |  |
| setName(String name) | broad.core.motif.PositionWeightMatrix |  |
| setUnparsedRow(int colNum, String[] unparsedRow) | broad.core.motif.PositionWeightMatrix |  |
| trimByInformationContent(double ic) | broad.core.motif.PositionWeightMatrix |  |
| write(BufferedWriter bw, NumberFormat formatter) | broad.core.motif.PositionWeightMatrix |  |
| writeUNIPROBE(BufferedWriter bw, NumberFormat formatter) | broad.core.motif.PositionWeightMatrix |  |


---

Generated on Fri Aug 15 2014 15:10:54 for ESAT by  

 1.8.7
